# Violence in War and Armed Conflicts as Experienced by Older Persons: A Meta Ethnographic Study

**DOI:** 10.1177/23333936251390441

**Published:** 2025-10-31

**Authors:** Elisabeth Lindberg, Maria Claesson, Åsa Israelsson-Skogsberg

**Affiliations:** 1 University of Borås, Sweden

**Keywords:** older persons, experiences, violence, war, armed conflicts, meta-ethnography, qualitative research

## Abstract

Older persons often stay in conflict zones, abandoned by younger generations and neglected by the government, putting them at risk of becoming victims of violence. This meta-ethnographic study aims to review and synthesise qualitative research on violence in contexts of war and armed conflicts as experienced by older persons and explore how violence in war and armed conflicts affects the health and well-being. Databases (CINAHL, PsychINFO, Web of Science, and Scopus) were searched for studies with a qualitative approach and participants aged ≥ 55 years. Twenty qualitative studies were included, describing experiences of persons from seven countries. *Guarding the past and ensuring a future* was established as an overarching metaphor in a lines-of-argument synthesis, accompanied by five themes: *To endure a violent situation; Home - the heart of existence; To witness a fragile family line; Alienated and abandoned by society- adding insult to injury* and *Maintaining normality in an abnormal situation.* Through interpretation, an understanding emerges of how separation from loved ones, the breakdown of healthcare services, and remaining in conflict areas can significantly increase vulnerability, while simultaneously demonstrating the resilience of older persons and their willingness to serve as resources within their communities.

## Introduction

Worldwide, people encounter violence in areas affected by war and armed conflicts. Despite the rules of war declared in the Geneva Conventions (United Nations, 1949), civilians are impacted as targets of violence. Conflicts within societies contribute to war-like situations, such as mass shootings, gang-related violence and civil war-like conditions. Unresolved conflicts between countries affect people’s lives by posing a constant threat of potential violence that can erupt at any time. Older persons are particularly vulnerable; they are not exempt from violence, torture, rape or other severe mistreatment during wars or armed conflicts (Sleap, 2022). Older persons face specific challenges as they are often abandoned when younger generations leave, neglected by their governments and forgotten in the global discourse on conflicts (Kokorelias et al., 2023). Older persons can moreover experience difficulty in accessing humanitarian aid (Kar, 2022) and can sometimes be bypassed by aid organisations (Kokorelias et al., 2023). Research in this area is of central importance to better understand the experiences of violence in war and armed conflicts from older persons’ perspectives (Weisbrod & Lev-Wiesel, 2019). Therefore, this study aims to review and synthesise existing research on violence in contexts of war and armed conflicts as it is experienced by older persons. The study also aims to explore how violence in war and armed conflicts affects the health and well-being of older persons.

## Background

Living in a society under the threat of violence caused by war and armed conflicts affects the health and well-being of those involved. The theoretical foundation of this study is caring science based on phenomenology (Galvin & Todres, 2013), the rationale being that violence as a phenomenon needs to be examined from a first-person perspective, in this case, the older person who experiences living in proximity to war and armed conflict. Here, violence is defined according to the World Health Organization (WHO), which states: “*the intentional use of physical force or power, threatened or actual, against oneself, another person, or against a group or community, which either results in or has a high likelihood of resulting in injury, death, psychological harm, maldevelopment, or deprivation*” (World Health Organization, 2002). This project does not address domestic violence or self-inflicted violence as war is defined as organised and violent conflicts between countries or conflicts between separate groups within a country (civil wars) (UCDP, 2025). Nevertheless, the concept “armed conflicts” is chosen to include conflicts that fall outside the definition of war but highlight a closeness to armed violence, for example, gang-related or lethal violence that affects the life of ordinary citizens (International Committee of the Red Cross, 2016).

Caring science, based on phenomenology, is grounded in Husserl’s life-world theory (Husserl, 1970) and theory of intentionality (Husserl, 1975). Merleau-Ponty (2014) further developed Husserl’s life-world theory and described how existence and the body are interconnected. Drawing on Merleau-Ponty’s philosophy of the lived body, Staudigl (2013) describes a phenomenological understanding of violence as a lived experience. The vulnerability caused by violence extends beyond the physical body (exemplified in assault, torture, and rape) to experiences of violence in the form of exclusion and discrimination. Staudigl (2013, p. 18) concludes, “*It is not only through my physical body that I experience violence against me. The violation that violence gives rise to is conveyed as an experience of violence through a symbolic and semantic shaping that does not need to originate from a physical source*” (Staudigl, 2013).

Ageing changes the body, both biologically and in terms of lived experiences. Research has shown that older persons may be left behind or choose to remain in war areas, exposing them to the risk of becoming targets of war crimes (Kokorelias et al., 2023). During the first 2 years following Russia’s full-scale invasion of Ukraine, increased vulnerability for older persons compared to the population as a whole has been indicated (Office of the United Nations High Commissioner for Human Rights, 2023). War and armed conflicts disproportionately affect older women, older persons living in remote areas and those with severe frailty related to physical or cognitive disabilities (Karunakara & Stevenson, 2012; Massey et al., 2017). Hearing loss, vision impairment and cognitive decline have an impact in stressful situations (Simmons & Swahnberg, 2021), which affects older persons’ ability to reach a safe place in a potentially threatening situation (Manor et al., 2023; Office of the United Nations High Commissioner for Human Rights, 2023; Rozenblat & Iecovich, 2013). Older persons remaining in conflict areas are furthermore at high risk of being injured or killed by landmines (Office of the United Nations High Commissioner for Human Rights, 2023).

From a phenomenological perspective, violence in the context of war and armed conflict encompasses lived experiences that result in profound suffering for those affected. This meta-ethnographic study is based on the perspective that both objective (the actual situation of living amid violence due to war and armed conflicts) and subjective definitions (the individual’s experience of the situation) need to be understood simultaneously. Research shows that factors such as age and gender contribute to increased vulnerability, and older persons may choose to remain in conflict areas, compounding their vulnerability due to their deteriorating physical or cognitive abilities. Their situation risks being overshadowed in a global situation characterised by war and armed conflicts and given that older persons constitute a large part of the population in many countries and that the knowledge base in this area is weak, there is a need for further research.

## Aim

The aim is to review and synthesise existing research on violence in contexts of war and armed conflict as experienced by older persons. The study also aims to explore how violence in war and armed conflict affects the health and well-being of older persons.

## Method

A meta-ethnographic approach based on Noblit and Hare (1988) was chosen. Conducting studies in situations of war and armed conflict is challenging (Ellor & Mayo, 2018); a meta-ethnographic study is therefore appropriate as it synthesises previous research and deepens knowledge through interpretation (France et al., 2019). The eMERGe meta-ethnography reporting guidance (France et al., 2019) was used for transparency, and the study is registered in PROSPERO (Prospective register for systematic reviews) https://www.crd.york.ac.uk/PROSPERO/view/CRD420251051171.

Noblit and Hare (1988) describe seven overlapping phases through which a synthesis of existing literature can be conducted. *Getting started (phase 1).* The Population, Exposure, Outcome (PEO) search strategy (Bettany-Saltikov & McSherry, 2024) was adapted to clarify the main concepts and to structure the systematic database search. A research question was therefore formulated: *What are the lived experiences (O) of persons aged 55 and older (P) who have been exposed to violence in war and armed conflicts? (E).*

### Deciding What is Relevant (Phase 2)

The search was conducted in the databases CINAHL, PsychINFO, Web of Science, and Scopus on the 12th of May 2025, with no limitations on years. A university librarian supported in the search process. The search terms were violence in society OR ( armed conflict or war ) (All Fields) and (older adults or elderly or geriatric or geriatrics or ageing or senior or seniors or older people or aged 55 or 55+) (All Fields) and ( qualitative research or qualitative study or qualitative methods or interview) (All Fields) not Domestic violence (All Fields) and Review Article (Exclude—Document Types). Inclusion and exclusion criteria are presented in Table 1.

**Table 1.** Inclusion and Exclusion Criteria.

Inclusion criteria	Exclusion criteria
1. Participants aged ≥ 55 years with experiences of violence in war or armed conflicts in relation to health and wellbeing^a^ 2. Studies that use a qualitative approach or mixed method studies that include a qualitative component suitable for extraction 3. Peer-reviewed publication 4. Studies published in English language	1. Participants aged < 55years. 2. Studies focusing on domestic violence. 3. Studies that do not present original qualitative data 4. Invalid article types (e.g., conference abstracts, dissertations, and commentaries). Publications that are not peer-reviewed full journal articles (e.g., conference abstracts, dissertations, theses, commentaries, or editorials) 5. Studies with a quantitative approach.

a In areas affected by war and armed conflicts, 50 years is an appropriate age for definition (Karunakara & Stevenson, 2012). Considering this, the term “older” in this study include persons ≥ 55 years.

**Figure 1.**
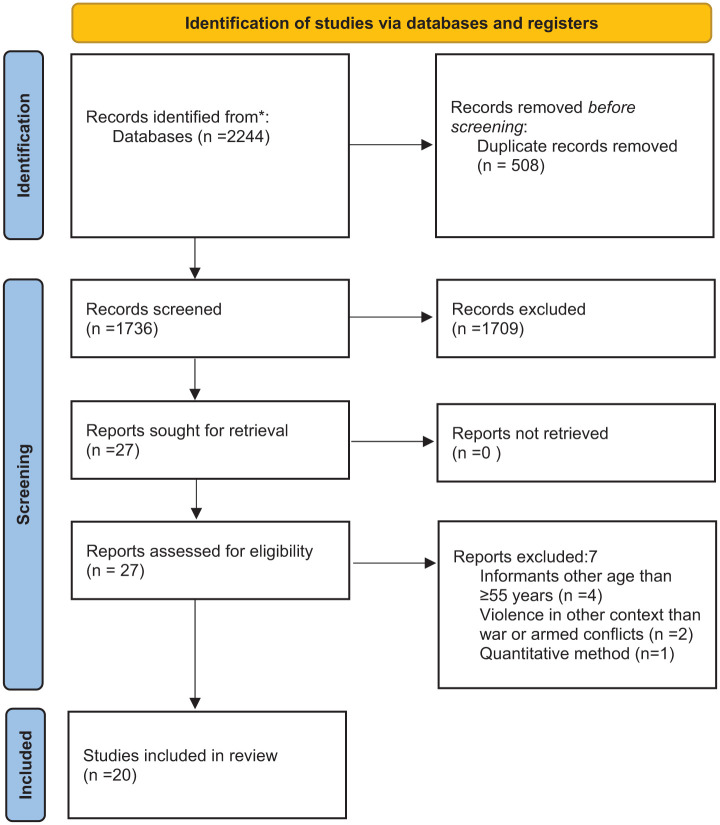
PRISMA diagram (Page et al., 2021).

The reference list of the included articles was reviewed to identify potential relevance. The guidelines for Preferred Reporting Items for Systematic Review and Meta-Analyses (PRISMA) were used to ensure transparency in methodology (Figure 1).

To assess the quality of the included articles, the checklist from the Critical Appraisal Skills Programme (CASP, 2024) for qualitative studies was used (Table 2).

**Table 2.** Critical Appraisal Skills Programme (CASP) Checklist for Qualitative Research (Critical Appraisal Skills Programme, 2024).

Study	Question									
	1	2	3	4	5	6	7	8	9	10
Apak et al. (2023)	Y	Y	Y	Y	Y	Y	Y	U	Y	Y
Arage et al. (2024)	Y	Y	Y	Y	Y	U	Y	Y	Y	Y
Carmel et al. (2016)	Y	Y	Y	Y	Y	Y	Y	Y	Y	Y
Chaitin et al. (2013)	Y	Y	Y	Y	Y	Y	U	Y	Y	Y
Curcio et al. (2019)	Y	Y	Y	Y	Y	Y	U	Y	Y	Y
Dubus (2018)	Y	Y	Y	Y	Y	Y	U	Y	Y	Y
Francis (2018)	Y	Y	Y	Y	Y	Y	Y	Y	Y	Y
Giebel, Saldarriaga-Ruiz et al. (2025)	Y	Y	Y	Y	Y	Y	Y	Y	Y	Y
Giebel, Montoya et al. (2025)	Y	Y	Y	Y	Y	Y	Y	Y	Y	Y
Hadida-Naus et al. (2023)	Y	Y	Y	Y	Y	Y	Y	Y	Y	Y
Nuttman-Shwartz and Regev (2018)	Y	Y	Y	Y	Y	Y	Y	Y	Y	Y
Nuttman-Shwartz and Spector-Mersel (2024)	Y	Y	Y	Y	Y	Y	Y	Y	Y	Y
Possick (2015)	Y	Y	Y	Y	Y	Y	U	Y	Y	Y
Regev and Nuttman-Shwartz (2019)	Y	Y	Y	Y	Y	Y	Y	Y	Y	Y
Shelef and Spector-Mersel (2025)	Y	Y	Y	Y	Y	Y	Y	Y	Y	Y
Singh et al. (2018)	Y	Y	Y	Y	Y	Y	Y	Y	Y	Y
Strug et al. (2004)	Y	Y	Y	Y	Y	Y	U	Y	Y	Y
Taei et al. (2023)	Y	Y	Y	Y	Y	Y	Y	Y	Y	Y
Tung et al. (2018)	Y	Y	Y	Y	Y	Y	U	Y	Y	Y
Ulitsa and Ayalon (2025)	Y	Y	Y	Y	Y	Y	Y	Y	Y	Y

*Note.* Y = Yes, N = No, U = Unclear. Critical appraisal questions: (1) Was there a clear statement of the aims of the research? (2) Is a qualitative methodology appropriate? (3) Was the research design appropriate to address the aims of the research? (4) Was the recruitment strategy appropriate to the aims of the research? (5) Was the data collected in a way that addressed the research issue? (6) Has the relationship between researcher and participant been adequately considered? (7) Have ethical issues been taken into consideration? (8) Was the data analysis sufficiently rigorous? (9) Is there a clear statement of findings? (10) Was this research valuable?.

### Reading Included Studies (Phase 3)

The included studies were read multiple times to gain a sense of their contents. All text was seen as contributing to the overall understanding, which means that the results, as described by each study’s authors (second-order constructions) and in the form of quotes (first-order constructions), were included (France et al., 2019). Table 3 provides an example of the data extraction process.

**Table 3.** Data Synthesis and Examples of First, Second and Third Orders and of Meaning Units.

Article	First order	Second order	Concepts/meaning units identified in phase 4	Third order (exemplified through the line of argument)
Arage et al (2023)	*“It is difficult to mention the hardship we passed. It was like living in a dark room without the knowledge of where to go. There was no water for drinking or electricity for cooking. There was no food to eat, as there were no millhouses and no market days, and the price was beyond the capacity of the community to pay.”* 60-years-old participant (Arage et al., 2023, p. 4).	The study shows that the conflict had inflicted a grim consequence to the health condition of the community, affecting individuals, families, and entire communities, with lives torn apart and supply chains disrupted (Arage et al. (2024, p. 4).	Lack of basic human supplies. Being in a situation of despair and suffering involving all aspects of life.	When basic and healthcare needs are compromised due to violence, older adults often face involuntary exile from active life, characterised by inactivity and loneliness. In addition, it causes a void between generations in which family lines are at risk of being broken.
Giebel, Saldarriaga-Ruiz et al. (2025)	*“You are afraid to talk to young people” (female 86* *years old) Giebel*, Saldarriaga-Ruiz et al., *2025, p. 4)*	Many older adults were complaining about the lack of respect experienced from younger generations, both within their own family and externally from their community. (Giebel, Saldarriaga-Ruiz et al., 2025 p. 4)	Being marginalised and mistreated.	
Possick (2015)	*“*That is *the revenge, that I have great-grandchildren and I have a family”* (Possick, 2015, p. 222).	The Holocaust was Leah’s formative experience in the development of her worldview; it is her framework for perceiving events and constructing their meaning. Throughout the interview, she speaks the language of war: victory, revenge, defeat (Possick, 2015, p. 222).	Revenge	Survival, in this context, becomes a form of resistance against the pervasive violence, and safeguarding the legacy of a long life becomes crucial for their well-being, and is perceived as a gift to generations to come

The included studies were published between 2003 and 2025 representing seven countries: Colombia, Ethiopia, Georgia, Israel, Syria, USA and Sweden. Some were from various age perspectives or used mixed methods, and only the perspectives of older persons and qualitative components were analysed to maintain consistency with the focus of the research question. This diversity in designs made it impossible to summarise the exact characteristics of the participants. The characteristics of the included studies are presented in Table 4.

**Table 4.** Included Articles.

Number	Author	Title	Aim	Participants	Setting	Method	Themes	Ethical approval
1	Apak et al. (2023)	*Evaluation of bio-psycho-social and socio-cultural problems of Syrian elderly living in Turkey.*	The aim was to determine the economic, biopsychosocial, and socio-cultural problems of the older Syrians living in Turkey	19 older persons migrants were interviewed. 10 of them were male and 9 were female. The mean age of the participants was 66.2 ± 7.10 (Min: 60; Max: 91	Syrian refugees in Turkey	Convergent mixed design was used. A semi-structured interview form was used to collect data in the interview	Experienced traumatic events, changing economic situation, and family relationships affected both physical and mental health of the older persons	This study was conducted by Bingöl University Ethics Committee approval was obtained.
2	Arage et al. (2023)	*Exploring the health consequences of armed conflict: the perspective of Northeast Ethiopia, 2022: a qualitative study.*	To explore the health consequences of the northern Ethiopia conflict with the aim of highlighting the burden of these problems from the perspective of affected individuals and communities.	100 respondents, among which 23 were persons >45 years.	Northeast Ethiopia, 2022	Interviews Focus groups Thematic analysis	Socio-demographic characteristics Health status of the community during the Northern Ethiopian conflict Mechanisms through which the conflict affected community health The devastating consequences of conflict on the health system Conflict elicited health challenges and coping mechanisms utilised that enhanced resilience	The study was conducted in accordance with the ethical declaration of Helsinki for research on human participant, after obtaining ethical approval from the Institutional Review Board (IRB) of Woldia University. Written informed consent was obtained from each respondent.
3	Carmel et al. (2016)	*Influences of Nationalism and Historical Traumatic events on the Will-to-Live of elderly Israelis*.	The aim of this study was to reveal additional factors to the personal factors already reported, which strengthen and/or weaken the will to live of older adults living in a relatively young country under constant existential threat.	25 Israeli citizens (16 women and 9 men), aged 75 and older (range 75–93)	Different areas of Israel including cities and kibbutzim	Semi-structured interviews Grounded theory	Two categories: (1) nationalism, (2) historical traumatic events.	Ethic approval were obtained from the Research Ethics Board of the Ben-Gurion University of the Negev.
4	Chaitin et al. (2013)	*“I May Look 75, but I’m Really a Pioneer”: Concept of Self and Resilience Among Israeli Elder Adults Living in a War Zone.*	How do elder adults from a war zone see themselves and how does this self-conception connect to resilience in the face of the ongoing security threat?	35 persons (19 women and 16 men) aged 65+	Eshkol, Israel close to the Gaza border.	Interviews and analysis with a biographical approach	13 overall themes were found: (1) the importance of family relations, (2) life’s emotional hardships, (3) economic difficulties, (4) social-political traumas, (5) life here versus there, (6) life’s opportunities or misses, (7) anti-Semitism or discrimination in the Diaspora, (8) being Jewish among individuals who grew up outside of Israel, (9) interpersonal violence, (10) wandering versus having a stable home, (11) the importance of Jewish immigration to Israel, (12) Zionism, and (13) the importance of being a pioneer.	Before the interview, each participant read and signed a consent form.
5	Curcio et al. (2019)	*Elderly and forced displacement in Colombia.*	To describe the experiences of older adults around forced displacement due to the Colombian armed conflict.	12 people aged over 60 years	Colombia	Interpretive-comprehensive study, with a hermeneutical approach	The narratives are presented in four sections: between indignity and survival; the drift of older people; displacement opens space but reduces existence, and between anguish and fear.	Participants signed an informed consent. Data was anonymised.
6	Dubus (2018)	*Arriving old: A qualitative study of elder refugee women’s self-perceptions of the first year of resettlement*	The study examines eight elder women’s experiences of resettling with their family and the protective factors that enhanced their resiliency.	8 Women aged 66–78 years.	Syrian refugees settled in Iceland	Interviews Thematic analysis	Three main themes: (1) before arriving in Iceland they felt their age was a liability to their families; (2) once in Iceland, they felt they had returned to a pre-civil war level of functioning; (3) witnessed their family continuing to struggle.	Before the interview, each participant read and signed an informed consent.
7	Francis (2018)	*A Narrative Inquiry Into the Experience of Being a Victim of Gun Violence.*	The purpose was to gain a greater understanding of gun violence from the victim’s personal story.	Sixteen victims of gun violence aged 18–71 years (Only results from persons ≥ 55 years of age are included in the present study.)	USA	Narrative inquiry. Victims of gun violence were interviewed and asked to tell their story	Four themes emerged: prevailing nature of everyday violence; feeling abandoned by the institutions of society; living in a context of reactive violence fuelled by poverty; lack of employable skills and education; and evolving psychological effect following gun violence.	Approval was obtained from the hospital’s institutional review board. Prior to the interviews, the research question and the purpose of the study were discussed with the potential participants. Opportunity was given to the participants to ask questions pertaining to the study.
8	Giebel, Saldarriaga-Ruiz et al. (2025)	*Coping in the Face of Violence – a Qualitative Study on the Impacts of Stressful Life Events on the Mental Health of Older Adults in Colombia.*	The aim was to understand the impact of stressful life events on the mental health and wellbeing of older Colombians living in areas of relative poverty.	26 older adults (aged 60+). 14 men and 12 women. Aged 62–96 years.	Turbo region in Colombia	Interviews Thematic analysis	Four overarching themes: (1) Living in violent and dangerous communities; (2) Disturbing gender violence and gender roles; (3) Lack of mental health awareness; (4) Coping mechanisms.	Ethical approval was obtained prior to study begin from the Universidad de Antioquia Ethics committee of the National Faculty of Public Health.
9	Giebel, Montoya et al. (2025)	*Addressing unmet mental health needs of older adults in Turbo, Colombia: a multi-component psychosocial intervention feasibility study*	The aim was to feasibility test a co-produced community-integrated intervention for older adults to improve their mental health and well-being in Turbo, Colombia.	Qualitative interviews with 12 participants (9 women and 3 men)	Turbo region in Colombia	Reflexive thematic analysis	Participants valued the intervention and the activities it offered, that the intervention was feasible, and expressed a keen interest for the intervention to be continued.	Ethical approval was sought from the Research Ethics Committee of the National Faculty of Public Health, University of Antioquia, prior to the intervention and data collection
10	Hadida-Naus et al. (2023)	*Alone in the Shadow of Terror: Coping Strategies and Internal Resources of Older Adults Living Alone in a Continuous Traumatic Situation.*	The study aimed to exploring how older adults living alone in continuous traumatic situations cope with challenges of ageing and loneliness, and the internal resources that help them do so.	15 persons aged 65+ years living (8 women and 7 men)	Sderot, Israel	In depth Interviews conducted and analysed thematically	Various coping strategies were described, such as positive thinking, deliberate disengagement, perception of being alone as an advantage. Internal resources that helped them cope: functional independence, faith, character traits, and previous experience with stressful life events.	The study was approved by the Ethics Committee of the Paul Baerwald School of Social Work and Social Welfare at the Hebrew University. All participants signed informed consent forms.
11	Nuttman-Shwartz and Regev (2018)	*Life in a continuous traumatic situation: perspective of the older population.*	The aim was to examine self-perceptions of coping patterns among older residents of rural communities near the Israeli border with Gaza during the Israel– Hamas war in the summer of 2014.	43 older persons (aged 65+)	Israel	Focus group interviews. Thematic analysis.	Four main themes: (1) moral issues: questions about leaving their place of residence or staying in the area; (2) emotional issues: the fluctuation between fear, “abandonment versus courage,” and maintaining a routine; (3) intergenerational issues: continuity versus change in generations; and (4) resilience: maintenance, containment and responsibility, as well as consideration of the future	Approval from the regional welfare department. All participants were informed and written invitations to an open meeting were distributed to older residents in the region
12	Nuttman-Shwartz and Spector-Mersel (2024)	*Cumulative Trauma: The Intersection Between Continuous Security Threats and the COVID-19 Pandemic Among Israeli Older Adults.*	To explore older adults’ perceptions of cumulative trauma, specifically exposure to continuous security threats via living in a war zone and COVID-19.	Seventeen (12 women and 5 men)	Israel	In-depth, open ended, and semi structured interviews. Thematic analysis.	The characteristics, difficulties, and emotions that accompanied each of the traumas revealed three trajectories: negative cumulative; positive cumulative; and unrelated.	The research was approved by Sapir College’s Board of Ethics.
13	Possick (2015)	*Grandparents’ Meaning Construction of the Loss of a Grandchild in a Terror Attack in Israel.*	The aim was to explore the personal and collective meanings that are given by grandparents to the death of their children in a terror attack.	12 grandparents (age 56–81 years) who had a grandchild killed in a terror attack in Israel.	Israel	Individual interviews. Textual analysis.	Two main categories. Loss in the personal context Loss in the collective context	Informed consent was obtained from all the interviewees.
14	Regev and Nuttman-Shwartz (2019)	*Coping styles and Aggregate Coping Styles: Responses of Older Adults to Continuous Traumatic Situations.*	The aim was to explore the variety of coping styles that older people use in the face of an extreme threat to their safety during wartime.	43 participants aged 65 and over (29 women and 14 men)	Israel	Focus groups. Analysed with deductive content analysis	The findings are presented by type of coping style Using a single coping style Using multiple coping styles	The researchers requested approval for the study from the regional welfare department. All participants voluntarily consented to participate.
15	Shelef and Spector-Mersel (2025)	*How to survive? Civilian women’s coping strategies in wartime captivity.*	The study aimed to analysing the strategies employed by civilian women of different ages to deal with the hardships of captivity.	17 oral interviews. Six of the study participants were women aged 63–85. (Only results from persons ≥ 55 years of age are included in the present study.)	Israel	17 oral interviews with the released abductees broadcast on Israeli television after their release.	The participants described various coping strategies, which were divided into three types: emotional; cognitive and behavioural	The study was approved by the Ethics Committee of the School of Social Work, Sapir College.
16	Singh et al. (2018)	*Identifying mental health problems and Idioms of distress among older adult internally displaced persons in Georgia.*	The study aimed to assess community-wide social and health problems with a focus on mental health problems and healthy functioning, as well as terminology used to describe these problems.	75 interviews with older adults above 60 years of age (44 women and 31 men). Aged 60–94 years	Georgia	Interviews with older adults. Thematic analysis.	The most frequently mentioned problems were related to health and economics. Psychosocial, distress, and displacement problems. Mental health and psychosocial problems included descriptions of potential illness symptoms relationships, and social roles.	The study protocol, consent forms, and instruments were reviewed by U.S. and Georgian institutional review boards (IRBs) in accordance with standard research procedures
17	Strug et al. (2004)	*An Exploratory Study of the Impact of the Year of 9/11 on Older Hispanic Immigrants in New York City*	The purpose was to document the reaction of a group of older Hispanic immigrants to a unique series of disasters	31 elderly (18 women and 13 men) Hispanics attending a community senior centre day programme	USA	Six focus groups were conducted	Most informants had recovered from their acute distress reactions to 9/11 and Flight 587 four months post-September 11, but many still experienced a wide range of psychological reactions related to these traumatic events, including anxiety, avoidance, and hypervigilance.	The researchers described the purpose of the study to prospective study participants in Spanish. They explained that participation was voluntary, and that the information collected would remain confidential.
18	Taei et al. (2023)	*Crime, Disorder, and Territorial Stigmatization: Older Adults Living in Deprived Neighbourhoods.*	This study explores how older adults in deprived neighbourhoods in Sweden experience crime and disorder, and how they adapt and respond to these problems and the neighbourhood’s poor reputation.	22 older adults (16 female and 6 men)	Sweden	Semistructured interviews with older adults who had lived 5 years or more in deprived areas of two cities in Sweden. Thematic analysis.	Most residents had positive things to say about their homes and neighbourhoods, even if criminal acts such as shootings, drug dealing, arson, burglary, and knife attacks were part of everyday life. The residents attempted to manage these events with various strategies.	All respondents gave written consent before the interview. The Swedish Ethical Review Authority approved the study.
19	Tung et al. (2018).	*Experiences of Community Violence Among Adults with Chronic Conditions: Qualitative Findings from Chicago.*	The purpose was to explore and characterise self-described experiences of community violence among adults with chronic health conditions.	The overall sample included 51 participants, ages 35 years and older, who had at least one chronic condition. (Only results from persons ≥ 55 years of age are included in the present study.)	USA	Individual and focus groups interviews. Grounded Theory	Major themes emerged: (1) perceived risk of being targeted, (2) chronic stress and worry, (3) hypervigilance, (4) social breakdown, (5) chronic isolation, (6) constrained choice (loss of freedom), (7) limited access to material resources, and (8) inadequate healthcare responses.	Data were extracted from audio recordings, transcribed verbatim, and de-identified during the transcription process.
20	Ulitsa and Ayalon (2025)	*“I worry about you more”: insights from older care recipients’ experiences during the Israel-Hamas war.*	The aim was to answer two research questions: 1) What are the main concerns of older care recipients during wartime? 2) What are their coping strategies for managing these concerns and worries?	13 older individuals with a mean age of 91.33 years (75% women and 25% men)	Israel	Interviews Analysed with thematic analysis	Two main themes were identified: 1) Concerns for others affected by war and concerns for oneself, and 2) Different coping strategies expressed by interviewees to address these concerns	Ethical consideration: Bar-Ilan University’s ethics committee approved the study. All interviewees signed an informed consent form prior to participating in the study.

### Determining How Studies Are Related and Translating Studies Into One Another (Phases 4 and 5)

The studies were related to each other by exploring meaning units in the content. A meaning unit is text contributing to a description and further development of knowledge in relation to the present study’s aim. The analysis proceeds by identifying recurring meaning units and by relating meaning units from the different studies. In phase 5, meaning units from each study were compared for similarities and differences; this included a search for related metaphors. An example is presented in Table 3, where the respondent says: “*It was like living in a dark room without the knowledge of where to go”* (Arage et al., 2024, p. 4). Here the metaphor “dark room” was interpreted as being in a situation of despair and suffering involving all aspects of life.

### Synthesising Translations and Expressing the Synthesis (Phases 6 and 7)

The studies are here viewed as a “whole” with the goal of developing a framework (Sattar et al., 2021). This resulted in a line of argument synthesis, which aims to provide a new interpretation, which became evident as the descriptions highlighted different perspectives of the phenomenon (Sattar et al., 2021). The synthetisation was developed by moving back and forth between the original data and the extractions, with the goal of constructing an interpretation representing something more than the parts alone, which are referred to as third-order constructions (Britten et al., 2002). During phase 7, the common themes across the studies were summarised by placing first and second-order constructions side by side for comparison. Third-order constructions were further developed by reading the primary data synthesis exemplified in Table 3 (Noblit & Hare, 1988; Sattar et al., 2021).

## Results

The characteristics of the included studies are presented, followed by five key themes: To endure a violent situation; Home—the heart of existence; To witness a fragile family line; Alienated and abandoned by society- adding insult to injury, Maintaining normality in an abnormal situation and the line of argument Guarding the past and ensuring a future; Resilience and contribution despite increased vulnerability.

## Characteristics of the Included Studies

The studies were conducted in Colombia, Ethiopia, Israel, Syria, USA, Georgia and Sweden representing a range of conflicts. The nature of the conflicts ranges from armed conflicts (e.g., terrorist attacks or gang related conflicts in Colombia, Sweden, or the USA) to violent conflicts between countries or conflicts between separate groups within a country (civil wars) as seen in Ethiopia, Syria and Georgia. The Israeli context lies somewhere between these descriptions, characterised by a constant threat of violence fluctuating between armed conflict and full-scale war.

## Themes

### To Endure a Violent Situation

War and armed conflict lead to deteriorated access to healthcare and a shortage of medical supplies, posing a potential risk to health. Physical- and existential wounds arise from witnessing disproportionate and brutal violence, and the memory of previous violent situations remains in the lived body. Fear and anxiety keep the entire being on alert: “*I jump at every noise*” (Regev & Nuttman-Shwartz, 2019, p. 165). Violence such as shootings and bomb explosions is interpreted as being in the middle of a nightmare (Arage et al., 2024) or “*on a volcano*” (Regev & Nuttman-Shwartz, 2019, p. 165). You never know what the next moment will bring, and basic activities like taking a shower mean lowering your guard for a few minutes and being exposed to potential danger (Nuttman-Shwartz & Regev, 2018). Moreover, as ageing can lead to changes in vision, hearing, mobility, and cognitive functions, the ability to seek shelter in a safe place during a threatening situation is affected (Hadida-Naus et al., 2023; Ulitsa & Ayalon, 2025).

Being a victim of violence in war and armed conflict involves facing the challenge of meeting basic humanitarian needs. The challenges vary from the direct lack of supplies such as food, water and medicine (Arage et al., 2024) or having to prioritise food over medicine (Giebel, Saldarriaga-Ruiz et al., 2025), to situations where the safety of moving around the local area makes it difficult to go to the store (Tung et al., 2018). Loss of appetite and sleep deprivation also occur (Strug et al., 2004), and when the local area is not safe, the ability to obtain nutritious food decreases (Curcio et al., 2019). The risk of malnutrition is not solely dependent on food availability but can be caused by anxiety and fear (Regev & Nuttman-Shwartz, 2019; Strug et al., 2004; Ulitsa & Ayalon, 2025). “*I lost seven kilograms. (. . .) I have no appetite. I can’t eat for a whole day. I do not want to eat. How can I eat after watching everything that happens on TV?*” (Ulitsa & Ayalon, 2025, p. 4).

Violence affects the possibility to make a living and can contribute to extreme poverty. To survive, food needs to be prioritised over care and medicine (Apak et al., 2023; Giebel, Saldarriaga-Ruiz et al., 2025). Lack of basic supplies and living in extreme poverty can result in a loss of hope for the future, where the meaningfulness of life is questioned. “*I was very sick, and my medicine was running out. This, combined with the starvation, put me in a stressed and desperate situation. Later, I lost all hope and began to wonder, ‘What is the meaning of life?*’” (Arage et al., 2024, p. 7). Vulnerability is also added to other challenges such as the Covid-19 pandemic. “*. . . at the end of our lives to suffer both from the disease and from terrorism?. . . Not easy. What can we do? How should we fight?”* (Nuttman-Shwartz & Spector-Mersel, 2024, p. 555).

Every aspect of life is affected when living near violence. Relationships with others may either deepen or become more distant. An existential loneliness can arise, which is exacerbated by the vulnerable situation (Tung et al., 2018; Ulitsa & Ayalon, 2025). Painful life experiences tend to become more apparent during war times: “A*fter my wife passed away, I went downhill on all levels. (. . .) I feel lonely. Especially now during this difficult time. . .I miss her so much. . . .*” (Ulitsa & Ayalon, 2025, p. 4). Loneliness can be painful, but it can also be a protection and a self-chosen strategy when living near violence and war. It is easier to seek shelter if you only have yourself to take care of, and when you are alone, you do not have to deal with others’ worries and anxieties. *“When there’s a red alert, they all get anxious [other persons] . . .I prefer to be alone at home. I see [them] shaking and crying, it makes me ill*.” (Hadida-Naus et al., 2023, p. 192). On the other hand, being among other persons such as a supportive family significantly impacts well-being: “*She [the granddaughter] comes over and cleans up. She sweeps, washes my clothes, and so on. I also have my son, who lives in the house opposite; he brings me food. He is always here. They are wonderful*” (Giebel, Saldarriaga-Ruiz et al., 2025, p. 5). Having a context to be in gives life meaning and strength and contributes to endurance (Giebel, Saldarriaga-Ruiz et al., 2025; Singh et al., 2018; Ulitsa & Ayalon, 2025).

### Home—The Heart of Existence

The choice between leaving the dangerous place or staying has a deep existential significance insofar as it entails being forced away from what is loved and familiar (Chaitin et al., 2013; Nuttman-Shwartz & Regev, 2018). The home is described as a place that should be protected and safe, a place where life can be lived. Even if there are opportunities to seek shelter elsewhere, it means becoming a stranger (Apak et al., 2023). There is a desire to end life in the place where life has been lived: “*I used to work in the garden. It is a beautiful place, and I prefer to die in this beautiful garden*” (Regev & Nuttman-Shwartz, 2019, p. 165). To maintain health and well-being, the least bad choice may be to stay in the familiar, and staying can be a way to show resilience (Chaitin et al., 2013; Nuttman-Shwartz & Regev, 2018) and to care for and protect what is important in the community: “*When people return, they need to see that they have a place to come back to*”(Regev & Nuttman-Shwartz, 2019, p. 166). When younger generations choose to leave, it causes conflicting feelings of understanding and sadness about the younger generations giving up their heritage too easily (Carmel et al., 2016; Nuttman-Shwartz & Regev, 2018).

When forced to leave, a situation of existential homelessness arises, where access to healthcare, medicine, food, and basic humanitarian support may be minimised. Older persons are at risk of assuming a self-inflicted burden by hindering younger relatives from leaving the violent area or causing trouble when there is a need to move quickly. “*We have such little time. And here I am needing to use the toilet. Not one, not two, but so many more times than they did. It is hard not to feel in the way when everyone stops for me.”* (Dubus, 2018, p. 399).

Being forced from home when living near violence and war, involves the loss of both an identity and a familiar world, one’s “cosmos” (Curcio et al., 2019), and can cause feelings of loneliness and abandonment (Nuttman-Shwartz & Regev, 2018,). Leaving risks becoming a dead end where violence continues and the existential homelessness is reinforced (Curcio et al., 2019; Nuttman-Shwartz & Regev, 2018; Singh et al., 2018). *“. . .the welcome they gave me was that the same night I arrived there, in Cali, they robbed me; there in . . ., (just) inside the city’s bus station, they stole my suitcase, the money I had, my mobile phone. . .*” (Curcio et al., 2019, p. 59). Older persons also experience violence simply because they are older, being chosen as victims of violence: “*I will not go into areas where I know I will be singled out. I keep a low profile. . . I am a senior, I have a condition where I need a cane, and I probably look like I can be pushed over quite easily*” (Tung et al., 2018, p. 1915).

### To Witness A Fragile Family Line

Violence causes tension between generations. When violence becomes widespread in a society, it is often perpetrated by the younger generation. This can lead to intergenerational tensions, and in a society where older persons must rely on family support, it can have long-term consequences, such as older persons being mistreated or abused: “*One is afraid to talk to young people*” (Giebel, Saldarriaga-Ruiz et al., 2025, p. 4). As a consequence of forced displacement, where different generations are compelled to live in close proximity under conditions of poverty and insecurity, intergenerational tensions become a significant burden on the situation for older persons (Singh et al., 2018).

Older persons also take responsibility for younger generations by questioning and intervening in situations when they, for example, witnessed drug dealing or offensive behaviour, situations that may be perceived as threatening (Taei et al., 2023); they watch over younger generations in threatening situations (Shelef & Spector-Mersel, 2025; Taei et al., 2023) and feel concern for them (Giebel, Saldarriaga-Ruiz et al., 2025; Strug et al., 2004). Being older means being a role model even in difficult situations, which can manifest itself as engaging with the youth in the local area (Giebel, Montoya et al., 2025; Taei et al., 2023), and carrying a desire for children and grandchildren, to be proud of them. An example of the latter is the story of an older woman who was a hostage in a violent situation: “*Thanks to my children, knowing and seeing that I am there for them, that I do not break. . . Be proud of me. . . whether I come back or not. I did not break.*” (Shelef & Spector-Mersel, 2025, p. 7).

Being forced to witness family members (including children) and friends being humiliated and raped causes wounds that deeply affect the older person (Arage et al., 2024). Historical gender roles make older women more vulnerable and exposed to violence and contribute to extreme poverty (Giebel, Saldarriaga-Ruiz et al., 2025) . Vulnerability can be related to being a woman, and in the hostage situation mentioned above, older women describe how they watched over a young girl in the group: “*We feel a great responsibility to take care of xx, a 17-year-old girl; we always had our eyes on her. We constantly tried [to ensure] she was not alone with the kidnappers*” (Shelef & Spector-Mersel, 2025, p. 9).

The well-being of children and grandchildren occupies their thoughts, and the fear is often greater in relation to their safety (Carmel et al., 2016; Ulitsa & Ayalon, 2025). Concern for loved ones affects health and well-being (Carmel et al., 2016). Having lived a long life becomes a shield against fear in relation to one’s own existence. This metaphorical shield is composed of a range of strategies, from holding on to hope for a better future to sheer defeatism (Shelef & Spector-Mersel, 2025; Ulitsa & Ayalon, 2025). Family and friends become the object of fear, and when they are injured or even killed, an existential abyss arises. The future of children and grandchildren is a concern (Ulitsa & Ayalon, 2025), and the family line can be broken when violence causes the death of younger generations. The grief over a lost grandchild is compounded by the grief of the mourning parents’ loss. Witnessing a grandchild’s death means that the life story has a dividing line, a before and an after (Giebel, Saldarriaga-Ruiz et al., 2025; Possick, 2015). “*See, what affected me most was the murder of my grandchild. . . he was with me everywhere, he was a very good son, a very good grandchild*” (Giebel, Saldarriaga-Ruiz et al., 2025, p. 7).

### Alienated and Abandoned by Society- Adding Insult to Injury

Violence in war and armed conflicts not only create wounds and despair for individuals but also generate collective experiences and trauma (Possick, 2015; Ulitsa & Ayalon, 2025). “*The attack happened to “I” and “us” but the loss is “general”, collective.”* (Possick, 2015, p. 224).

Violence challenges essential societal institutions such as healthcare and social services. If there is low governmental support, the risk of mistreatment increases even more as institutions, healthcare and social services can be taken over by warring parties or subjected to corruption (Arage et al., 2024; Giebel, Saldarriaga-Ruiz et al., 2025). Healthcare facilities run by combatants are perceived as less reliable, deprioritising health and care of the civilian population, which has long-term consequences affecting the vulnerable older population. “*After they took over the hospital, they told us to go and get treatment, but who would have the courage? Who knows what they will provide? What if they poison us?”* (Arage et al., 2024, p. 6). When healthcare institutions collapse, older persons are left with no choice but to take care of themselves or turn to religious communities, which then become arenas for support and comfort (Giebel, Saldarriaga-Ruiz et al., 2025).

The burden on societal institutions increases as supply chains break down (Arage et al., 2024), and shops and banks tend to be absent in areas with high violence and crime. For older persons living in these areas, this means difficulties in making purchases and conducting banking transactions, and it also deprives them of the meeting place that a vibrant city centre can provide: “*If you have to post a letter, you have to take the bus to the centre*!” (Taei et al., 2023, p. 4).

There are situations when trust turns into mistrust. Such situations can occur when authorities force you to leave or resettle “*How could this be my future? How could this be my life? I am old. I believed I would live my final years at home*” (Dubus, 2018, p. 400), or when violence occurs after you have been told that the danger is over (Nuttman-Shwartz & Regev, 2018), or when there is a lack of presence of societal protective agencies such as the police (Francis, 2018). The lack of follow-up from healthcare is described as adding to a feeling of being abandoned by societal institutions (Giebel, Saldarriaga-Ruiz et al., 2025; Tung et al., 2018).

### Maintaining Normality in an Abnormal Situation

Older persons show strength and determination and contribute to maintaining important functions in society during times of war and armed conflicts. Taking care of properties and looking after (grand)children are examples of contributions to society, as is being involved in community organisations and supporting society with what they can. Distributing food and medicine are examples of life-saving initiatives where older persons act as role models, instil hope and encourage others to support the community (Arage et al., 2024; Regev & Nuttman-Shwartz, 2019). Other examples include engaging in rescue teams (Regev & Nuttman-Shwartz, 2019) and maintaining the ability to be self-sufficient (Hadida-Naus et al., 2023). Additional strategies involve continuing to live life as normally as possible (Giebel, Saldarriaga-Ruiz et al., 2025; Regev & Nuttman-Shwartz, 2019) , not putting too much effort into emotional aspects (Giebel, Saldarriaga-Ruiz et al., 2025), and maintaining relationships with children, grandchildren and friends (Giebel, Montoya et al., 2025; Giebel, Saldarriaga-Ruiz et al., 2025; Regev & Nuttman-Shwartz, 2019; Ulitsa & Ayalon, 2025).

Having lived through previous wars and conflicts means having experiences that are important to share with younger generations (Giebel, Montoya et al., 2025; Hadida-Naus et al., 2023; Nuttman-Shwartz & Regev, 2018) and contributing to resilience (Hadida-Naus et al., 2023; Ulitsa & Ayalon, 2025). Ageing is seen as a privilege (Giebel, Saldarriaga-Ruiz et al., 2025, p. 4): “*For me, it is a pleasure, it is a luxury, that God allows one to reach this age because young people do not even reach 20, they destroy themselves*.” Being a survivor of previous wars can be interpreted as vindication and revenge against those who subjected you to trauma (Carmel et al., 2016; Possick, 2015): “*That is the revenge, that I have great-grandchildren and I have a family*” (Possick, 2015, p. 222). However, past trauma is also revived and affects the experience of what is happening here and now (Apak et al., 2023; Strug et al., 2004). Being a survivor can create a sense of guilt (Shelef & Spector-Mersel, 2025) or strengthen the belief that the current situation will also pass and that you will get through it (Ulitsa & Ayalon, 2025).

Resistance lies in the spirit of trying to maintain as much normality as possible. Focusing on daily activities but having a plan for how to handle potentially violent situations becomes a way to show resilience (Regev & Nuttman-Shwartz, 2019), and it is important to maintain dignity (Hadida-Naus et al., 2023; Shelef & Spector-Mersel, 2025). Various creative activities such as writing (Hadida-Naus et al., 2023; Shelef & Spector-Mersel, 2025), engaging in dialogue (Shelef & Spector-Mersel, 2025), or taking part in community-based activities (Giebel, Montoya et al., 2025) are used as distractions.

Being exposed to violence can cause a fatalistic attitude where one’s own death is not the most frightening option. Examples are when caregivers are encouraged to leave the older person behind and seek safety for themselves (Ulitsa & Ayalon, 2025), or when danger is challenged by confrontation, as exemplified by woman choosing to resist her fear by opening the door to her house during a rocket attack: “*Instead of crying, I opened the door. I was afraid, but I did it. . .)”*(Nuttman-Shwartz & Regev, 2018, p. 961). Letting fear and emotions pass—mainly due to a lack of other options—is described as a way to continue in life (Giebel, Saldarriaga-Ruiz et al., 2025). Dying from some form of violence is also described as a way to have a painless death without prolonged suffering (Nuttman-Shwartz & Spector-Mersel, 2024).

### Line of Argument: Guarding the Past and Ensuring a Future; Resilience and Contribution Despite Increased Vulnerability

A compelling line of argument has been developed, encapsulated by the overarching metaphor: Guarding the past and ensuring a future. This metaphor underscores the resilience and contributions of older adults, even amidst heightened vulnerability.

In contexts marked by violence, older persons demonstrate remarkable resilience through active engagement in the welfare of their communities. Their contributions during times of turmoil provide them with a sense of meaning and purpose. The act of self-care and maintaining independence fosters a sense of pride in their abilities and achievements. Survival, in this context, becomes a form of resistance against the pervasive violence, and safeguarding the legacy of a long life becomes crucial for their well-being and is perceived as a gift to generations to come.

Resilience and contribution persist despite the looming threat of increased vulnerability. When basic and healthcare needs are compromised due to violence, older adults often face involuntary exile from active life, characterised by inactivity and loneliness. In addition, it causes a void between generations in which family lines are at risk of being broken. The failure to recognise and value the resources that older persons bring is not merely a personal issue; it is a significant problem for the state, society, and humanity at large.

## Discussion

In this study, we synthesised the experiences of violence in contexts of war and armed conflict as lived by older individuals and examined how such violence impacts their health and well-being. The line of argument—*Guarding the past and ensuring a future: resilience and contribution despite increased vulnerability*—is grounded in the understanding that proximity to violence affects older people’s opportunities for health and well-being and causes a void between generations. At the same time, it evokes resilience in the shape of courage, engagement and a will to live to assist in the process of create possibilities for future generations. The findings of the present study closely align with the central themes of Frankl’s theses in “Man’s Search for Meaning” (1985). The significance of finding meaning amid a horrifying situation serves as the “why” in life, which can overcome “how” the present situation has evolved. This concept, as articulated by Frankl and originally derived from Nietzsche, posits that “*Those who have a ‘why’ to live, can bear with almost any ‘how*’” (Frankl, 1985, p. 76). In the spirit of finding a *why* older persons are never just recipients of help in violent situations, they are part of the solution (Ellor & Mayo, 2018). They have the potential to contribute and participate productively to society either by paid work or by civic engagement and non-economic contributions to their families. Older persons have much to offer as they have long experiences of life events which can contribute to problem solving and managing potential conflicts (van Den Hoonaard, 2018). Human beings are transcending, living and moving. Returning to Frankl (1985), this possibility to transcend is grounded in a freedom to choose how to live life even under the most extreme circumstances. Freedom can, for example, be to tend the garden, which aligns with previous research highlighting the importance of nature for older adults (Magnussen et al., 2021). Freedom can also be to confront fear during a rocket attack, resisting fear and refusing to hide and cry (Nuttman-Shwartz & Regev, 2018, p. 961).

Older persons are not spared from suffering in violent situations, as the results indicate they can be specific targets for violence. Violence leads to both physical and existential wounds, and when access to care is affected, the ultimate purpose of care—to alleviate suffering and create conditions for health and well-being—is not achieved. The need for governmental and societal support is crucial, as research indicates that societies that have lost the ability to care for and nurture their most vulnerable citizens through, for example, corruption and neglect within public services cause significant damage to older persons’ access to care, thereby affecting their health and well-being. This issue is documented in countries such as Colombia , which experience internal conflicts; it has also emerged as a problem in Sweden, where criminality has infiltrated the welfare sector (Government of Sweden, 2025). During periods of violence, structural barriers such as logistical challenges, damage to infrastructure, and staff shortages due to conflict present significant difficulties for older adults. This results in reduced access to care facilities (van Boetzelaer et al., 2025). Furthermore, when health care personnel are under constant strain and experience, for example, sleep disturbances (Alqaissi et al., 2025), shortages of staff and overwork lead to exhaustion and compromise the quality of patient care (van Wyk & Naicker, 2023).

Every situation marked by violence has its own background and character, and the older persons in these situations experience them under unique conditions. The variations exist on a continuum where escape is possible and preferred to where escape is neither possible nor desirable. Regardless of whether the older person stays or leaves the conflict area, the home remains the epicentre, soul and heart of existence. In areas with longstanding and ongoing conflicts, such as the Israeli Palestinian conflict, a transgenerational traumatic experience is formed, meaning that the experience of violence in war and armed conflicts is passed to younger generations. Grandparents experiences of violence becomes part of the children’s games and is transformed into a new generation (Veronese et al., 2021). This transformation is conducted through storytelling, which can also have a central role in maintaining cultural and more family-specific rituals in situations of displacement (Atallah, 2017). As interpreted in the results, the family line becomes increasingly fragile in times of violence. The loss of family members can take different forms, and the fear of losing loved ones is almost unbearable and a foundation for grief. For an older person, remaining alive while younger family members are injured or killed represents a profound paradox of life. When younger generations are involved in violence (violent gangs, drug dealing, etc.) a situation can occur where the younger person choose violence over family ties. Regardless of the nature of the loss, these experiences deeply affect the older persons wellbeing and become part of their lived experiences. As Brinkmann (2018) describes, grief is a fundamental human experience, deeply intertwined with our capacity to love. To grieve is to remain emotionally connected to the person who has been lost, often for the rest of one’s life—making grief a profound existential concern.

Being a victim of gun-violence (despite age and gender) is a severe trauma, and the wounded body is a body wounded as an entire being physiologically, psychologically and existentially. To heal is a process in which dimensions such as gaining trust towards the society also needs to be included (Francis, 2024). To support health and wellbeing in times of violence, education is of importance for preparedness. In Sweden, for example, there is a lack of education targeting older persons in situations of disasters (Andersson et al., 2024), and older persons also need to be acknowledged in specific support programmes (Kazberouk & Sinha, 2023). It is crucial to consider the impact of past trauma, as these experiences can affect the well-being of older persons, particularly persons with cognitive impairments (Craftman et al., 2020).

### Methodological Considerations

Titles and abstracts have been screened, and it would have been laudable to have more research from a wider range of areas exposed to violence in war and armed conflicts. This geographic imbalance is a limitation in terms of generalisability. However, qualitative studies focusing older persons’ experiences in vulnerable situations such as war or armed conflicts or humanitarian crisis are sparse (Massey et al., 2017). One reason is that the possibilities to conduct research in violent areas are limited as taken for granted aspects of research, such as having access to digital devices and an infrastructure for storing collected data, can be hindered. The researcher and the informants share an equal risk of being harmed or inconvenienced in relation to the combatants or governments (Höglund & Öberg, 2011), and when approaching older persons in extremely vulnerable situations, the researcher needs to have a sensitivity for ethical aspects (Akram, 2021).

Given the substantial efforts dedicated to researching the circumstances of older persons in this specific context, studies employing a meta-ethnographic approach are valuable. They foster a new, potentially deeper understanding through the synthesis of previous studies. In meta-ethnography, the phases are intertwined; the synthesis is affected by an evolving understanding taking place during the process (Noblit & Hare, 1988). The line of argument with the metaphor *Guarding the past and ensuring a future; Resilience and contribution despite increased vulnerability*, contributes, as described by Noblit and Hare (1988), to illuminated hidden meanings and formulated conclusions of relevance in a clinical context. The interpretative approach in meta-ethnography contributes to abstraction of the results from the included studies in that way underlines transferability to other geographical areas and other contexts in which the situation of vulnerable people needs to be considered. A limitation of this study lies in the level of interpretation achieved in the results. The study addresses a complex phenomenon, drawing on articles from a diverse range of conflicts and cultural contexts. Striking a balance between remaining faithful to the study’s aim and presenting findings in a clear and accessible manner is inherently challenging. We have endeavoured to follow the guidelines set out by Noblit and Hare (1988) to the best of our ability; however, this may have influenced the depth of interpretation.

The informants in the included articles were ≥55 years. Old age differs across the world and depends on demographic and geographical locations. Instead of defining it in relation to years lived, it can also be defined in relation to stages in life (Overall, 2016), as in the article by Possnick (2015) in which the focus is on “grandparents” rather than a defined age. The articles in the dataset also included studies with mixed-method approaches and voices from persons <55 years, but only the perspectives of persons ≥55 years and qualitative results were included. More research is needed in this area concerning the most vulnerable older persons, such as those with cognitive impairment, women or those in great need of medical and health support.

## Conclusions

This project started as a response to the lack of reports on the impact that situations of violence caused by war and armed conflict have on older persons. It also started as a response to preconceptions underpinned by an ageist attitude in which older persons are perceived as a burden to society, a pre-understanding that can easily be consolidated and strengthened in relation to situations in which older persons are exposed to violence. Being old in a violent society inflicts on life and threatens close relationships and family ties. Simultaneously, the results provide knowledge with strong arguments suggesting that instead of being a burden on society, older persons contribute in many ways. By being carriers of history, they help to strengthen cultural anchoring and maintain cultural heritage. They also help to provide care to other people and to the place where they live. There is a pride in the stories of older persons where the desire to be independent and not lose hope is central. However, for a society to take advantage of and protect the resources older persons bring to society, a trusting and strong social contract is needed where the state and society take responsibility for maintaining opportunities for caring and welfare even in vulnerable situations.
